# Clinical impact of endocapillary proliferation with modified cutoff points in IgA nephropathy patients

**DOI:** 10.1371/journal.pone.0214414

**Published:** 2019-03-29

**Authors:** Ahmad Baseer Kaihan, Yoshinari Yasuda, Takahiro Imaizumi, Koji Inagaki, Takaya Ozeki, Manabu Hishida, Takayuki Katsuno, Naotake Tsuboi, Shoichi Maruyama

**Affiliations:** 1 Department of Nephrology, Nagoya University Graduate School of Medicine, Nagoya, Japan; 2 Department of Nephrology/CKD initiatives, Nagoya University Graduate School of Medicine, Nagoya, Japan; Peking University First Hospital, CHINA

## Abstract

Predictive values of mesangial proliferation (M), endocapillary proliferation (E), segmental glomerulosclerosis (S), and crescents (C) among 19 validation studies of the Oxford Classification of IgA nephropathy (IgAN) were discrepant, especially in Asian patients. These validation studies indicate that cutoffs of MESC score in the Oxford Classification may not be generalizable. Thus, we aimed to improve the clinical value of MESC scores by modifying the cutoff points. A total of 104 patients with IgAN were diagnosed from 2001 to 2012 vai renal biopsy and retrospectively evaluated at Nagoya University Hospital. The cutoff point for modified (M´E´S´C´) was determined using the receiver operating characteristic curve in association with renal outcome in the training cohort. Clinical values of the Oxford MESTC vs M´E´S´C´ cutoff points were analyzed using Kaplan-Meier and Cox regression in association with poor renal outcome in the validation and the entire cohort. Of 104 patients, 12.5% reached poor renal outcome over a median of 6.25 [4.16–9.61] years of follow-up. The modified cutoffs were defined as ≥40%, ≥10%, ≥20%, and ≥5% in the glomeruli for M´E´S´, and C´ respectively. In univariate analysis, E´, S ´, and T were significantly associated with poor renal outcome, whereas Oxford MESC, M´, and C´ in the training and validation cohort were not associated with poor renal outcome. Using multivariate analysis in the presence of estimated glomerular filtration rate (eGFR), only E´ was a significant predictive factor for poor renal outcome. The E´ with modified cutoff point of 10% significantly improved predictive value for poor renal outcome in IgAN. Therefore, the clinical value of modified cutoff points for M´E´S´C´ scores should be validated with various cohort studies in different regions.

## Introduction

IgA nephropathy (IgAN) is one of the most common glomerulonephritis, with a higher incidence in the Pacific Rim and Mediterranean countries.[1,2] Renal biopsy confirms the diagnosis of IgAN, assesses disease severity, and guides therapeutic strategies in clinical practice.[3] Although several pathological classifications have been developed from expert opinion, each has its limitations and none has achieved widespread agreement. [3–6] Therefore, the International IgA Network and Pathology Society developed the Oxford Classification of IgAN in a cohort of 265 patients with IgAN comprising mainly Caucasians, as only 20 and 28 adults with IgAN from Japan and China, respectively, were included. [6,7]

The original Oxford Classification study has demonstrated prognostic values of mesangial hypercellularity (M), endocapillary proliferation (E), segmental glomerulosclerosis (S), tubular atrophy/ interstitial fibrosis (T). Nineteen validation analyses, including the Oxford Classification, have been reported, of which 17 showed predictive value of T score. However, the prognostic value of S, M, E, and C were confirmed only in 8, 6, 3, and 0 validation studies, respectively. [8] In Japan, Katafuchi et al. reported the predictive value of T and C scores in adult patients with IgAN, [9] and our former study showed that only T score was associated with renal outcome.[10] Most of the validation studies from Asian countries could not demonstrate the predictive values of MESC score in the Oxford Classification.[10–16]

Both Katafuchi et al.’s and our previous study revealed a considerably smaller positive number of M and T scores and a higher positive number of E and C scores. In Japan, patients with IgAN seem to be diagnosed at an earlier phase because Japanese people undergo annual health examinations, including urinalysis.[17] Therefore, cutoff points of MESC in the Oxford Classification may not be optimal for Japanese patients with IgAN. Thus, in this study, we aimed to improve the clinical value of MESC scores by modifying their cutoff points.

## Materials and methods

### Study design and participants

**Fig 1:**
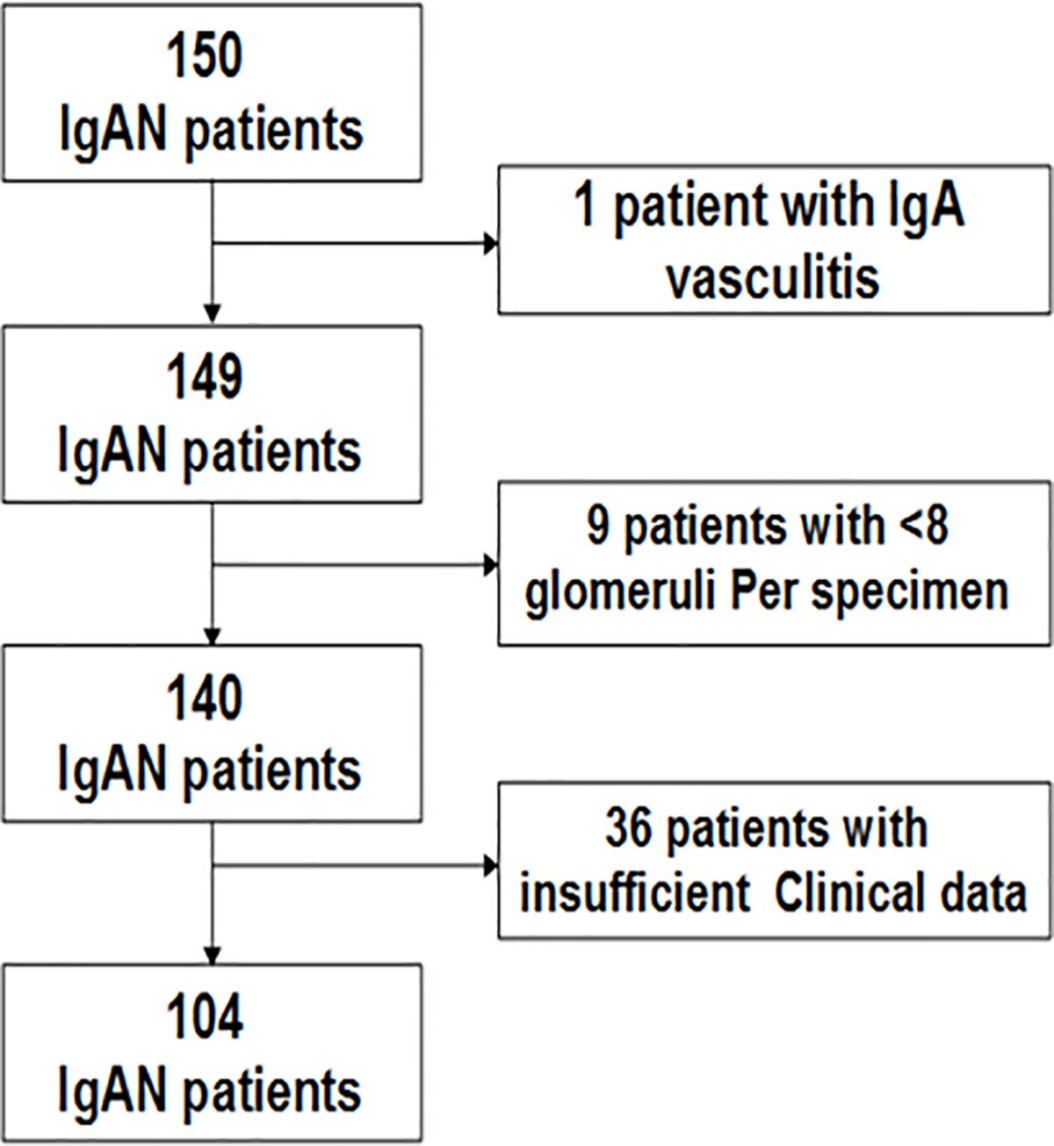
The flowchart shows the number of IgAN patients excluded from the cohort study.

This retrospective cohort study was conducted at the Nagoya University Hospital. All patients with IgAN were enrolled between 2001 and 2012. Patients with the following criteria (age ≥18 years, primary IgAN, follow-up period >1 year) and specimens with ≥8 glomeruli were included. Forty-six patients were excluded from this study (Fig 1), and the remaining 104 patients with IgAN were analyzed, including patients with <0.5 g/day proteinuria (n = 24) and advanced stage of disease (eGFR <30 ml/min per 1.73 m^2^_,_ n = 4). The ethics committee of the Nagoya University Hospital approved this retrospective study design without written informed consents, but the informed consent was obtained from almost entire patients with IgAN at the time of renal biopsy (IRB no. 1135/2015-0386).

### Definitions

The baseline was defined as the time of renal biopsy. The clinical variables measured at the time of renal biopsy were; age (yr), BMI (kg/m^2^), serum cholesterol (mg/dl), proteinuria (g/day), eGFR (ml/min/1.73m^2^). Hypertension was defined as systolic blood pressure (SBP) ≥140 mmHg and/or diastolic blood pressure (DBP), ≥90 mmHg and/or on antihypertensive therapy; estimated glomerular filtration rate (eGFR) was estimated using the Japanese GFR equation [= 194 x SCr ^-1.094^ x age ^-0.287^ x 0.739 (if female)] (SCr; serum creatinine).[18] The time interval between renal biopsy and the last outpatient visit, death, end-stage renal disease (ESRD) (eGFR <15 ml/min/1.73 m^2^) or initiation of hemodialysis during this time period was defined as follow-up period. RAS-blockade (ACE inhibitors and/or angiotensin receptor blockers) treatment described at any dose during follow-up and Immunosuppressive therapy was defined according to uniformed treatment regimens of Pozzi’s et al.[19] After renal biopsy. Renal outcome was defined as a 50% increase in SCr and/or initiation of hemodialysis (HD) from baseline. [2,10,20–22] Proteinuria and eGFR at the end of follow-up were also measured, and followed until the end of 2016.

### Renal biopsy review

All specimens were analyzed according to the Oxford Classifications [23]: mean M score was measured in all scorable glomeruli based on severity of cell proliferation per mesangial area (0–3), and the proportions of E1, S1, and cellular and fibro-cellular crescents (C1) in all obtained glomeruli were recorded in a scoring sheet. These histological characteristics blinded from the clinical data.

All specimens with a thickness of 2–3 μm were stained with periodic acid-Schiff, hematoxylin and eosin, periodic acid-methenamine silver, and Masson’s trichrome was assessed by AB.K and a group of expert nephrologist (YY, *TK*, TI, TO, MH, KI).

### Modified cutoff points

All 104 patients were divided into three equal sets: two-third of samples accounted for the training cohort and one-third of samples accounted for the validation cohort. A random selection was conducted according to the date of renal biopsy. The cutoff point for modified (M´E´S´C´) was determined using receiver operating characteristic curve (ROC) analysis. Youden Index[24] was used to obtain optimal cutoffs according to mean M score and the proportions of positive ESC scores in association with poor renal outcome in the training cohort. The clinical value of traditional MESTC vs M´E´S´C´ cutoff points were analyzed using Kaplan-Meier and Cox regression in association with poor renal outcome in the validation cohort.

### Statistical analysis

The clinical values of the Oxford MESTC cutoffs vs. modified M´E´S´C´ cutoff points were analyzed using Kaplan-Meier analysis and the significant difference in renal survival curve between the two groups was assessed using the log-rank test. The Cox proportional hazards model was used in the univariate and multivariate analyses in association with poor renal outcome in the training cohort, validation cohort, and in the entire cohort. Significant variables determined using univariate Cox-regression analysis and the clinical relevant factor (eGFR) was selected in multivariate Cox-regression models in all 104 patients. Results were expressed as hazard ratios (HR) with 95% confidence intervals (CI). The correlation between histological features and clinical variables was analyzed using the Spearman test.

Parametric and nonparametric variables were represented as the median and interquartile range [IQR]. The student’s test was used to analyze the difference in parametric variables. Categorical variables were depicted in percentages and compared using the chi-squared test. The proportional hazards assumption for covariates was tested using Schoenfeld residuals. P<0.05 was considered statistically significant. All statistical analyses were conducted using STATA/SE 14.2. (Stata Corp.2015, College Station, Texas, USA).

## Results

### Clinical characteristics

The patient’s baseline characteristics are shown in Table 1. Between the training and validation cohorts, significant differences were shown only in baseline eGFR and the total number of patients treated with RAS-blockade, but differences were not observed in other clinical variables.

**Table 1:** Baseline and follow-up clinical characteristics in the training and validation cohorts.

Baseline variables	Training cohort (n = 70)		Validation cohort (n = 34)		p value
	Median, no	IQR, (%)	Median, no	IQR, (%)	
**Gender (F)**	37	(53)	21	(62)	0.39
**Age (year)**	33	[24–45]	37	[28–46]	0.84
**BMI (kg/m^2^)**	21	[20–24]	22	[21–24]	0.701
**HT + (-)**	23/47	(33)/(67)	9/25	(27)/(73)	0.51
**SBP (mm-Hg)**	120	[107–129]	120	[108–130]	0.831
**DBP (mm-Hg)**	70	[63–80]	71	[64–82]	0.83
**TCH (mg/dl)**	205	[180–228]	198	[176–222]	0.181
**Serum IgA (mg/dl)**	300	[237–360]	326	[259–375]	0.89
**Proteinuria (g/day)**	0.96	[0.57–1.68]	0.83	0.49–1.43]	0.36
**SCr (mg/dl)**	0.8	[0.7–1.0]	0.9	[0.7–1.1]	0.911
**eGFR (ml/min/1.73m^2^)**	79	[59–95]	66	[55–83]	0.02*
**Follow-up**					
**Follow-up period (year)**	6.5	[4.2–9.8]	6.4	[4.2–9.6]	-
**Prednisone + (-)**	50/20	(71)/(29)	21/13	(62)/(38)	0.32
**RAS-blockade (ACEi/ARB) + (-)**	58/12	(83)/(17)	22/12	(65)/(35)	0.04*
**Underwent to tonsillectomy + (-)**	26/44	(37)/(63)	27-Jul	(21)/(79)	0.09
**SBP (mm-Hg)**	114	[106–129]	117	[102–124]	0.586
**DBP (mm-Hg)**	70	[60–80]	67	[60–72]	0.164
**Proteinuria (g/g Cr)**	0.34	[0.18–1.28]	0.31	[0.13–0.98]	0.798
**eGFR (ml/min-1.73m^2^)**	68.2	[44.1–85.7]	64.3	[45.4–81.8]	0.386

*IQR* interquartile range, *f* female, *BMI* body mass index, *SBP/DBP* systolic/ diastolic blood pressure, *TCH* total serum cholesterol, *IgA* Immunoglobulin A, *SCr* serum creatinine, *eGFR* estimated glomerular filtration rate.

*P<0.05: statistically significant

Of 104 patients, 12.5% reached poor renal outcome in over 6.25 [4.16–9.61] years of follow-up. The baseline proteinuria, SCr, and proportion of hypertension in this study vs. original Oxford Classification were (0.96 vs 1.7) g/day, SCr (0.8 vs 1.2) mg/dl, and (35 vs 65) %, respectively.

### Modified threshold

ROC analysis determined a mean M score of ≥38% as the optimal cutoff point for M’, and the proportions of ≥8%, ≥17%, ≥4% as optimal cutoffs for E´, S’, and, C´ respectively, in the training cohort. For the ease of clinical use, modified cutoff points of 40%, 10%, 20%, and 5% for M´, E´, S´, and C´ were defined, respectively. The chi-squared test demonstrated significant differences between proportions of M1 vs. M´1, E1 vs. E´1, S1 vs. S´1, and C1 vs. C´1 and the results are shown in Table 2.

**Table 2:** Numbers and proportions of positive MESTC scores according to Oxford and modified cutoff points in the entire cohort.

Pathologic features	Original Oxford MESTC	Modified M´E´S´C´	P value
**M1 no (%)**	22 (21)	38 (37)	<0.001*
**E1 no (%)**	37 (36)	18 (17)	<0.001*
**S1 no (%)**	78 (75)	24 (23)	0.001*
**C1 no (%)**	53 (52)	38 (37)	<0.001*
**^a^T1+2 no (%)**	17 (16)	-	-

*M´* modified mesangial hypercellularity, *E´* modified endocapillary proliferation, *S´* modified segmental glomerulosclerosis, *C´* modified crescents.

^a^ Cutoff for tubular atrophy and interstitial fibrosis in modified classification is the same as Oxford classification.

*p<0.05: Statistically significant by chi-squared test

### Univariate analysis

#### Kaplan-Meier analysis

**Fig 2:**
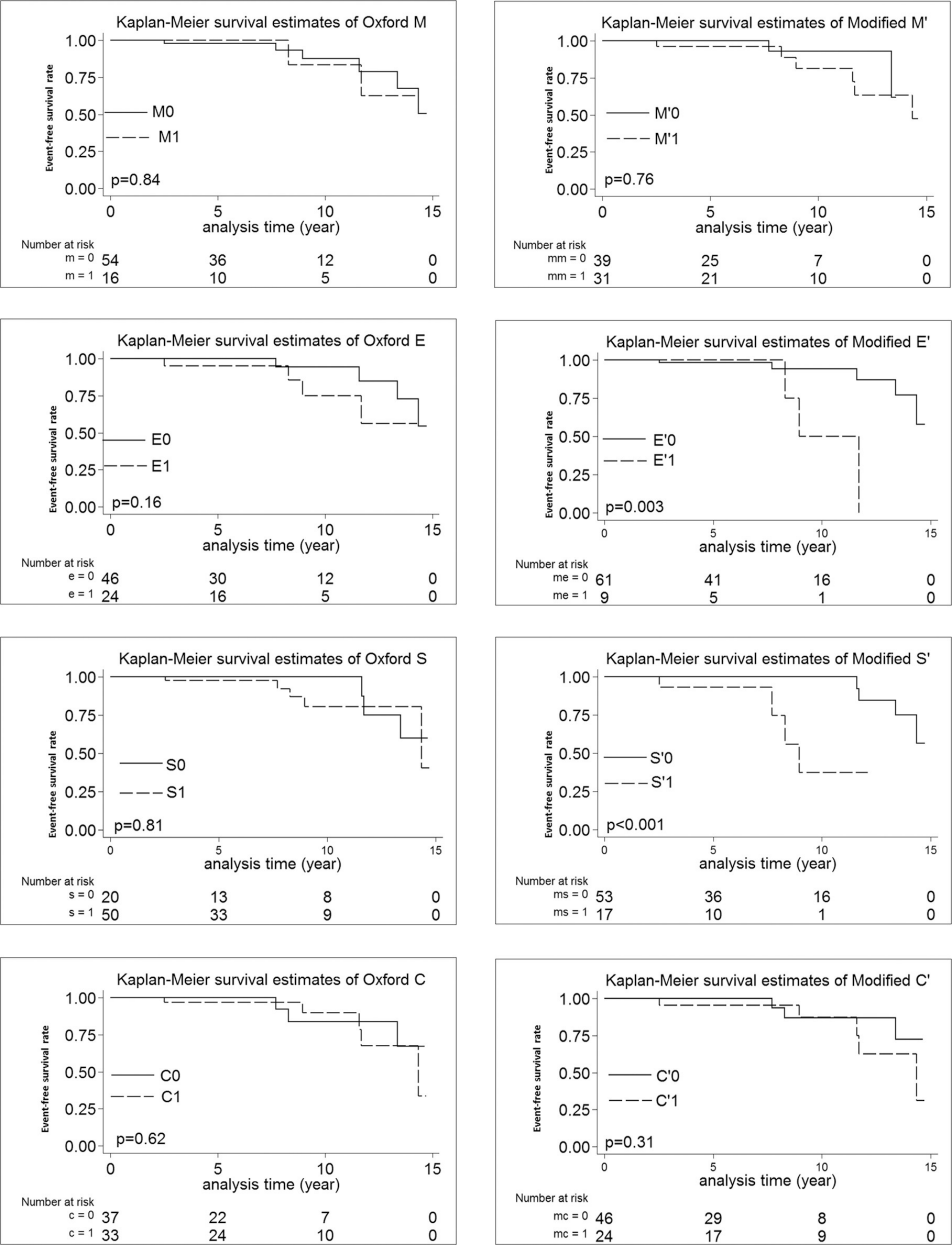
Kaplan-Meier analysis according to the original Oxford MESC vs. modified M´E´S´C´ cutoffs in the training cohort.

**Fig 3:**
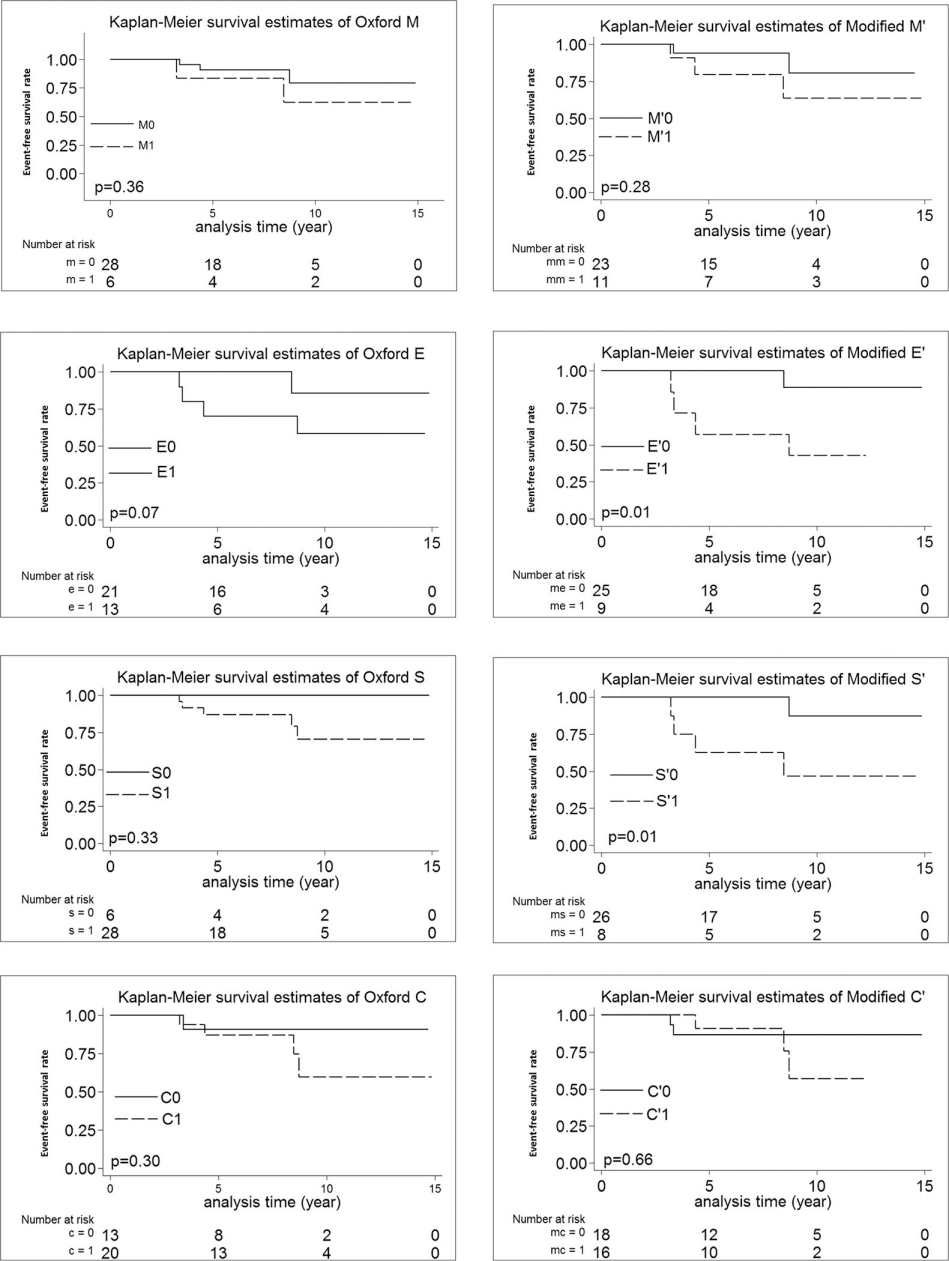
Kaplan-Meier analysis according to the original Oxford MESC vs. modified M´E´S´C´ cutoffs in the validation cohort.

Renal survival free from renal outcome was significantly lower in E´ (p = 0.01) and S´ (p<0.01) in the log-rank test in both training and validation cohorts (Figs 2 and 3).

#### Cox regression analysis

The risk of progression to poor renal outcome was significantly higher in E´ (HR = 7.98, p = 0.01) and S´ (HR = 13.67, p = 0.003); whereas, the Oxford MESC, M´, and C´ had no significant association with poor renal outcome in the training cohort (Table 3). Similarly, in the validation study, E´ (HR = 11.27, p = 0.03) and S´ (HR = 10.60, p = 0.003) demonstrated a significant risk of progression to poor renal outcome, but neither the Oxford MESC nor M´ and C´ cutoffs were associated with poor renal outcome (Table 3). Among the clinical variables, eGFR was significantly associated with poor renal outcome but not proteinuria.

**Table 3:** The hazard ratio of the Oxford MESTC and modified M´E´S´C´ scores for renal outcome in the training and validations cohorts.

**Training cohort**			
**Original Oxford cutoffs**	**Univariate HR (95% CI)**	**Modified M´E´S´C ´cutoffs**	**Univariate HR (95% CI)**
**M0 <50%**	Reference	M´0 <40%	Reference
**M1 ≥50%**	1.19 (0.23–6.16)	M´1 ≥40%	1.27 (0.27–5.87)
**p**	0.840	p	0.761
**E0 (absent)**	Reference	E´0 <10%	Reference
**E1 (present)**	2.85 (0.63–12.87)	E´1 ≥10%	7.98 (1.58–40.45)
**p**	0.171	p	0.010*
**S0 (absent)**	Reference	S´0 <20%	Reference
**S1 (present)**	1.32 (0.31–5.72)	E´1 ≥20%	13.67 (2.39–78.25)
**p**	0.710	p	0.003*
**C0 (absent)**	Reference	C´0 <5%	Reference
**C1 (present)**	1.44 (0.34–6.09)	C´1 ≥5%	2.09 (0.49–8.86)
**p**	0.620	p	0.320
**T0 (<25%)**	Reference	-	-
**T1+2 (≥25%)**	14.14 (3.07–65.04)	-	-
**p**	0.001*	-	-
**Validation cohort**			
**M0 <50%**	Reference	M´0 <40%	Reference
**M1 ≥50%**	2.28 (0.37–13.89)	M´1 ≥40%	2.57 (0.43–15.4)
**p**	0.371	p	301
**E0 (absent)**	Reference	E´0 <10%	Reference
**E1 (present)**	6.12 (0.67–55.74)	E´1 ≥10%	11.27 (1.24–102.21)
**p**	0.110	p	0.030*
**S0 (absent)**	Reference	S´0 <20%	Reference
**S1 (present)**	1.52 (0.29–7.98)	E´1 ≥20%	10.60 (1.18–94.99)
**p**	0.800	p	0.003*
**C0 (absent)**	Reference	C´0 <5%	Reference
**C1 (present)**	3.00 (0.33–27.02)	C´1 ≥5%	1.49 (0.25–8.93)
**p**	0.331	p	0.660
**T0 (<25%)**	Reference	-	-
**T1+2 (≥25%)**	6.87 (1.14–41.28)	-	-
**p**	0.034*	-	-

*HR* hazard ratio, *M*´ modified mesangial hypercellularity, *E*´ modified endocapillary proliferations, *S´* modified segmental glomerulosclerosis, *C´* crescents.

*p<0.05: Statistically significant.

In the multivariate model-I analysis, E´, S´, C´, and T scores were significantly associated with poor renal outcome. The significant pathological factors in univariate analysis, namely E´, S´, T, and eGFR were selected in the multivariate model-II analysis, which demonstrated that E´ (HR = 8.25, p = 0.004) and eGFR (HR = 0.96, p = 0.029) were significant factors for poor renal outcome (Table 4).

**Table 4:** Hazard ratio of covariates for renal outcome by Cox regression analysis in all 104 patients.

Variables	Univariate		Multivariate model-I		Multivariate model-II	
	HR (95% CI)	P value	HR (95% CI)	P value	HR (95% CI)	P value
**M´0/1**	1.78 (0.56–5.68)	0.330	2.04 (0.53–7.90)	0.300	-	
**E´0/1**	9.26 (2.69–31.95)	<0.001*	7.63 (1.93–30.19)	0.004*	8.25 (1.95–34.84)	0.004*
**S´0/1**	5.70 (1.88–17.33)	0.002*	8.79 (1.92–40.29)	0.005*	3.12 (0.80–12.08)	0.100
**T0/T1+2**	7.59 (2.46–23.41)	< 0.001*	9.82 (2.26–42.60)	0.002*	2.60 (0.68–9.99)	0.163
**C´0/1**	2.06 (0.67–6.32)	0.209	8.70 (1.40–53.86)	0.020*	-	
**Age**	1.01 (0.97–1.05)	0.530	-	-	-	
**Gender**	0.45 (0.15–1.38)	0.161	-	-	-	
**BMI**	1.04 (0.89–1.23)	0.610	-	-	-	
**HT**	2.27 (0.74–6.94)	0.150	-	-	-	
**eGFR**	0.95 (0.92–0.98)	0.002*	-	-	0.96 (0.93–0.99)	0.029*
**UPE**	1.33 (0.74–2.40)	0.334	-	-	-	

HR hazard ratio, *M´* modified mesangial hypercellularity, *E´* modified endocapillary proliferation, *S´* modified segmental glomerulosclerosis, *and C´ modified crescents*, *T tubular atrophy /interstitial* fibrosis, HT hypertension, UPE urine protein excretion.

*p<0.05: Statistically significant

Among clinical and pathological features, T score was significantly correlated with eGFR, proteinuria, and S´ score (Table 5).

**Table 5:** Correlations between modified M´E´S´C´ cutoffs, the Oxford T score, and clinical parameters.

variables		E´	S´	T	C´	proteinuria	eGFR
M´	rho	-0.03	0.01	0.01	0.70	0.41	0.97
	p	0.76	0.91	0.98	0.50	0.08	0.01*
**E´**	rho	-	0.05	0.24	0.04	0.29	0.16
	p	-	0.61	0.01*	0.70	0.003**	0.71
**S´**	rho	-	-	0.21	-0.03	0.19	-0.10
	p	-	-	0.03*	0.75	0.06	0.31
**T**	rho	-	-	-	-0.19	0.26	-0.30
	p	-	-	-	0.06	0.01**	<0.001**
**C´**	rho	-	-	-	-	0.18	0.05
	p	-	-	-	-	0.07	0.60

*M´* modified mesangial hypercellularity, *E´* modified endocapillary proliferation, *S´* modified segmental glomerulosclerosis, *and C´ modified crescents*, *T tubular atrophy /interstitial* fibrosis, rho Spearman's correlation coefficient

**^p^< 0.05. Correlation is significant at the 0.01 level (2-tail)

*^p^ <0.05. Correlation is significant at the 0.05 level (1-tail)*p <0.05. Correlation is significant at the 0.05 level (1-tail)

## Discussions

In this study, we proposed to improve the prognostic value of MESC scores in the Oxford Classification in association with poor renal outcome defined as a 50% increase in SCr and/or HD initiation from baseline. Our results showed that E´ (≥10% of glomeruli with E lesion), S´ (≥20% of glomeruli with S lesion) and T in both training and validation cohort were significantly associated with poor renal outcome. However, neither the Oxford MESC nor M´ and C´ scores demonstrated an association with poor renal outcome in both training and validation cohorts. Moreover, in multivariate analysis in the presence of E´_,_ S´, T, and baseline eGFR among all 104 participants, only E´ was an independent and significant histological factor for poor renal outcome. To best of our knowledge, this is the first study that analyzed modified thresholds of MESC score and validated them in an external cohort.

In the literature to date, only one study by Shi et al. has analyzed the threshold of E25 (25% of glomeruli with E1) but failed to reveal a significant association of E25 with ESRD.[25] The major differences between our study and that of Shi et al.’s were in definition of renal outcome and methods to set cutoff points. We defined renal outcome as 50% SCr increase and/or HD initiation, whereas Shi et al. only analyzed ESRD for renal outcome. The reason they set the threshold of E25 was not documented. Moreover, patients with mild stage of disease were excluded in Shi et al.’s study; therefore, the patients in Shi et al.’s study had more proteinuria (1.7 g/day vs. 0.9 g/day) than our cohort.

Among the pathological variables, E´, S´, C´, and T scores were significantly associated with poor renal outcome. However, in the presence of baseline eGFR with significant pathological variables in univariate analysis, only the E´ score was significantly associated with poor renal outcome. Furthermore, a significant correlation between T score and baseline eGFR was also demonstrated. Therefore, the association between T score and renal outcome was attenuated by adding eGFR, implying that the effect of baseline eGFR on the renal outcome was mediated by the interstitial changes. This result is in line with that from Alamartin et al.’s study.[26] In addition, T1+T2 was less frequent in our cohort study than in former studies, which might be because Japanese patients with IgAN were diagnosed in an earlier phase of the disease in our study. Stefan et al [27] reported that tubular atrophy/interstitial fibrosis and segmental sclerosis indicates an advanced stage of IgAN renal damage and their predictive power for renal survival is not surprising. Additionally, tubular atrophy and interstitial fibrosis are not the characteristic lesions of IgAN, and they are universal predictors of renal outcome among most chronic kidney disease patients.

The prognostic value of C´ in this study, could not be improved, which might be due to small sample size and only four patients having a baseline eGFR of < 30ml/min/1.73m^2^. We were unable to analyze C2 (≥25% of glomeruli with crescents) in association with renal outcome because only two patients had >25% of crescents in our cohort.

Even though 68.3% of IgAN patients in our cohort were treated with corticosteroids, the significant association of E´ with renal outcome was consistent. In the Oxford Classification study patients with positive E score were more likely to have been treated with immunosuppressive treatments including additional immunosuppressive agents in the follow-up, whereas no patients received cytotoxic agents in our study. Ballardie et al. reported the clinical effect of early combination therapy of corticosteroid with low dose cytotoxic immunosuppressive agents in a clinical trial study among IgAN patients.[28] Therefore, the treatment strategy for Japanese patients with IgAN should be reconsidered in the future.

This study has some limitations. First, it was a retrospective study design with a small number of participants. However, significant results were obtained in this study. Second, we determined the optimal cutoff points in a cohort of Japanese patients with IgAN, but these cutoff points may not be generalized to different regions and the cutoffs might change based on disease severity. However, we believe that the modified cutoff points for M´E´S´C´ would be better prognostic predictors the original Oxford Classification MESC cutoff points that were considered only in the presence and absence for ES and C and need to be validated in different regions with larger sample size. Third, we defined renal outcome as 50% increase in SCr from baseline, although doubling of SCr was analyzed as renal outcome in clinical trials. Several previous studies among patients with IgAN demonstrated the clinical significance of 50% increase in SCr level as a renal endpoint [2, 20–22].

In conclusion, the E´ with modified cutoff point of 10% significantly improved the predictive value for poor renal outcome in IgAN. The clinical value of the modified cutoff points for M´E´S´C´ scores should be validated with various cohort studies in different regions.

## Supporting information

S1 File This supporting file shows ROC-Tables and ROC Figures for modified M’E’S’C’. (XLSX)

## References

[1] Donadio J V, Grande JP. IgA nephropathy. N Engl J Med. 2002;347: 738–48. 10.1056/NEJMra020109 12213946

[2] Tatematsu M, Yasuda Y, Morita Y, Sakamoto I, Kurata K, Naruse T, et al Complete remission within 2 years predicts a good prognosis after methylprednisolone pulse therapy in patients with IgA nephropathy. Clin Exp Nephrol. 2012; 10.1007/s10157-012-0644-0 22618296

[3] Bartosik LP, Lajoie G, Sugar L, Cattran DC. Predicting progression in IgA nephropathy. Am J Kidney Dis. 2001;38: 728–735. 10.1053/ajkd.2001.27689 11576875

[4] Lee HS, Koh HI, Lee HB, Park HC. IgA nephropathy in Korea: a morphological and clinical study. Clin Nephrol. 1987;27: 131–140. 3552343

[5] Alamartine E, Sabatier JC, Berthoux FC. Comparison of pathological lesions on repeated renal biopsies in 73 patients with primary IgA glomerulonephritis: value of quantitative scoring and approach to final prognosis. Clin Nephrol. 1990;34: 45–51. Available: http://www.ncbi.nlm.nih.gov/pubmed/2225552 2225552

[6] Cattran DC, Coppo R, Cook HT, Feehally J, Roberts ISD, Troyanov S, et al The Oxford classification of IgA nephropathy: rationale, clinicopathological correlations, and classification. Kidney Int. 2009;76: 534–45. 10.1038/ki.2009.243 19571791

[7] Walsh M, Sar A, Lee D, Yilmaz S, Benediktsson H, Manns B, et al Histopathologic features aid in predicting risk for progression of IgA nephropathy. Clin J Am Soc Nephrol. 2010;5: 425–430. 10.2215/CJN.06530909 20089495 PMC2827572

[8] Trimarchi H, Barratt J, Cattran DC, Cook HT, Coppo R, Haas M, et al Oxford Classification of IgA nephropathy 2016: an update from the IgA Nephropathy Classification Working Group. Kidney Int. 2017;91: 1014–1021. 10.1016/j.kint.2017.02.003 28341274

[9] Katafuchi R, Ninomiya T, Nagata M, Mitsuiki K, Hirakata H. Validation study of oxford classification of IgA nephropathy: the significance of extracapillary proliferation. Clin J Am Soc Nephrol. 2011;6: 2806–2813. 10.2215/CJN.02890311 22157710 PMC3255377

[10] Kaihan AB, Yasuda Y, Katsuno T, Kato S, Imaizumi T, Ozeki T, et al The Japanese Histologic Classification and T-score in the Oxford Classification system could predict renal outcome in Japanese IgA nephropathy patients. Clin Exp Nephrol. Springer Japan; 2017;21: 986–994. 10.1007/s10157-017-1393-x 28349230

[11] Kang SH, Choi SR, Park HS, Lee JY, Sun IO, Hwang HS, et al The Oxford classification as a predictor of prognosis in patients with IgA nephropathy. Nephrology Dialysis Transplantation. 2012 pp. 252–258. 10.1093/ndt/gfr295 21606384

[12] Nasri H, Mortazavi M, Ghorbani A, Shahbazian H, Kheiri S, Baradaran A, et al Oxford-MEST classification in IgA nephropathy patients: A report from Iran. J Nephropathol. 2012;1: 31–42. 10.5812/jnp.7 24475384 PMC3886168

[13] Park KS, Han SH, Kie JH, Nam KH, Lee MJ, Lim BJ, et al Comparison of the Haas and the Oxford classifications for prediction of renal outcome in patients with IgA nephropathy. Hum Pathol. 2014;45: 236–243. 10.1016/j.humpath.2013.08.019 24439222

[14] Le W, Zeng C-H, Liu Z, Liu D, Yang Q, Lin R-X, et al Validation of the Oxford classification of IgA nephropathy for pediatric patients from China. BMC Nephrol. 2012;13: 158 10.1186/1471-2369-13-158 23181565 PMC3519602

[15] Shima Y, Nakanishi K, Hama T, Mukaiyama H, Togawa H, Hashimura Y, et al Validity of the Oxford classification of IgA nephropathy in children. Pediatr Nephrol. 2012;27: 783–792. 10.1007/s00467-011-2061-0 22134880

[16] Kfoury H, Alsuwaida A, Hussain S, Aloudah N, Alhejaili F, Alsaad K, et al External validation of the Oxford classification of IgA nephropathy: A retrospective study of 70 patients from Saudi Arabia Hong Kong J Nephrol. Elsevier Taiwan LLC; 2014;16: 29–33. 10.1016/j.hkjn.2014.04.001

[17] Kawamura T, Joh K, Okonogi H, Koike K, Utsunomiya Y, Miyazaki Y, et al A histologic classification of IgA nephropathy for predicting long-term prognosis: Emphasis on end-stage renal disease. J Nephrol. 2013;26: 350–357. 10.5301/jn.5000151 22684645

[18] Matsuo S, Imai E, Horio M, Yasuda Y, Tomita K, Nitta K, et al Revised Equations for Estimated GFR From Serum Creatinine in Japan. Am J Kidney Dis. 2009;53: 982–992. 10.1053/j.ajkd.2008.12.034 19339088

[19] Pozzi C, Bolasco PG, Fogazzi GB, Andrulli S, Altieri P, Ponticelli C, et al Corticosteroids in IgA nephropathy: a randomised controlled trial. Lancet. 1999;353: 883–887. 10.1016/S0140-6736(98)03563-6 10093981

[20] Peters HPE, Brand JAJ Van Den, Berger SP, Wetzels JFM. Immunosuppressive therapy in patients with IgA nephropathy.: 284–289. 26228193

[21] J.V. DJ, J.P. G, E.J. B, R.A. D, T.S. L, D.C. S. The long-term outcome of patients with IgA nephropathy treated with fish oil in a controlled trial. J Am Soc Nephrol. 1999;10: 1772–1777. 10446945 10.1681/ASN.V1081772

[22] Lee H, Hwang JH, Paik JH, Ryu HJ, Kim DK, Chin HJ, et al Long-term prognosis of clinically early IgA nephropathy is not always favorable. BMC Nephrol. 2014;15: 94 10.1186/1471-2369-15-94 24946688 PMC4070337

[23] Roberts ISD, Cook HT, Troyanov S, Alpers CE, Amore A, Barratt J, et al The Oxford classification of IgA nephropathy: Pathology definitions, correlations, and reproducibility. Kidney Int. 2009;76: 546–556. 10.1038/ki.2009.168 19571790

[24] Stefan M, Panduru NM, Ion DA. Estimation of youden index and its associated optimal cut-point when the parameters of gamma biomarkers are estimated by the method of moments. Proc Rom Acad Ser A—Math Phys Tech Sci Inf Sci. 2011;12: 269–276.

[25] Shi SF, Wang SX, Jiang L, Ji-Cheng L, Liu LJ, Chen YQ, et al Pathologic predictors of renal outcome and therapeutic efficacy in IgA nephropathy: Validation of the Oxford classification. Clin J Am Soc Nephrol. 2011;6: 2175–2184. 10.2215/CJN.11521210 21852672 PMC3358999

[26] Alamartine E, Sauron C, Laurent B, Sury A, Seffert A, Mariat C. The use of the oxford classification of IgA nephropathy to predict renal survival. Clin J Am Soc Nephrol. 2011; 10.2215/CJN.01170211 21885791 PMC3359557

[27] Ştefan G, Ismail G, Stancu S, Zugravu A, Andronesi A, Mandache E, et al Validation study of Oxford Classification of IgA Nephropathy: the significance of extracapillary hypercellularity and mesangial IgG immunostaining. Pathol Int. 2016;66: 453–459. 10.1111/pin.12442 27439692

[28] Ballardie FW, Roberts ISD. Controlled prospective trial of prednisolone and cytotoxics in progressive IgA nephropathy. J Am Soc Nephrol. 2002;13: 142–148. 11752031 10.1681/ASN.V131142

